# Mechanisms of peripheral levodopa resistance in Parkinson’s disease

**DOI:** 10.1038/s41531-022-00321-y

**Published:** 2022-05-11

**Authors:** Milan Beckers, Bastiaan R. Bloem, Marcel M. Verbeek

**Affiliations:** 1 Department of Neurology, Donders Institute for Brain, Cognition and Behaviour, Radboud University Medical Center, Nijmegen, The Netherlands; 2 Radboudumc Center of Expertise for Parkinson & Movement Disorders, Nijmegen, The Netherlands; 3 Department of Laboratory Medicine, Radboud Institute for Molecular Life Sciences, Radboud University Medical Center, Nijmegen, The Netherlands

**Keywords:** Parkinson's disease, Parkinson's disease, Parkinson's disease, Parkinson's disease, Neurodegeneration

## Abstract

Parkinson’s disease (PD) is an increasingly common neurodegenerative condition. The disease has a significant negative impact on quality of life, but a personalized management approach can help reduce disability. Pharmacotherapy with levodopa remains the cornerstone of treatment, and a gratifying and sustained response to this treatment is a supportive criterion that argues in favor of an underlying diagnosis of PD. Yet, in daily practice, it is not uncommon to encounter patients who appear to have true PD, but who nevertheless seem to lose the responsiveness to levodopa (secondary non-responders). Some patients may even fail to respond altogether (primary non-responders). Here, we address how two mechanisms of “peripheral resistance” may underlie this failing response to levodopa in persons with PD. The first explanation relates to impaired bowel motility leading to secondary bacterial overgrowth, and more specifically, to the excessive bacterial production of the enzyme tyrosine decarboxylase (TDC). This enzyme may convert levodopa to dopamine in the gut, thereby hampering entry into the circulation and, subsequently, into the brain. The second explanation relates to the systemic induction of the enzyme aromatic l-amino acid decarboxylase (AADC), leading to premature conversion of levodopa into dopamine, again limiting the bioavailability within the brain. We discuss these two mechanisms and focus on the clinical implications, potential treatments and directions for future research.

## Introduction

Parkinson’s disease (PD) is the second-most common neurodegenerative condition. The prevalence of the disease is growing faster than what could be explained by aging of the population alone. Consequently, the global number of PD patients is expected to more than double in the next twenty years, and might exceed 17 million by the year 2040^1^. The clinical presentation encompasses both motor and non-motor symptoms, leading to progressive disability and a marked reduction in quality of life. Fluctuations in the response to pharmacotherapy, which are common in persons with advanced disease, are a further source of quality of life reduction^2,3^.

A multidisciplinary management approach tailored to the needs of each individual can help reduce disability. Drug treatment is one of the four main pillars of this integrated approach, alongside device-aided therapies, multidisciplinary care and patient empowerment^4^. Pharmacotherapy with levodopa has remained the cornerstone of the overall treatment plan ever since its introduction in 1961^5^ and usually helps to improve activities of daily living as well as quality of life^6,7^. Indeed, a gratifying and sustained response to dopaminergic drugs is a supportive criterion that argues in favor of an underlying diagnosis of PD^8^. Yet, in daily practice, it is not uncommon to encounter patients who appear to have true PD, but who nevertheless do not respond adequately to levodopa. Some patients may fail to respond altogether, even when there is otherwise little doubt about the diagnosis of PD^9,10^. Many other persons with PD develop a progressive resistance to even adequately dosed levodopa treatment over time, despite having initially enjoyed a beneficial response. In tertiary referral centers such as ours, these persons can number as high as 20%, but this percentage may be inflated because patients with a poor response to levodopa have a greater likelihood of being referred to a tertiary center of expertise. Consequently, these percentages may well be lower in more general clinics, but we suspect that future research might show such a diminished response to be a considerable issue in the general PD population as well. In this viewpoint, we discuss two mechanisms of “peripheral resistance” that may underlie this failing response to levodopa in persons with PD. Identification of these patients with a peripherally blunted levodopa responsiveness may have important clinical implications, as it could avoid the typically prolonged and time-consuming process of gradual further levodopa dosage increases, and instead motivate a timely start of alternative, more effective treatment strategies (e.g., with dopamine receptor agonists). Other approaches can target the mechanism of peripheral resistance directly. Timely installment of such treatments could help to avoid unnecessary disability.

## Peripheral levodopa pharmacokinetics

**Fig. 1:**
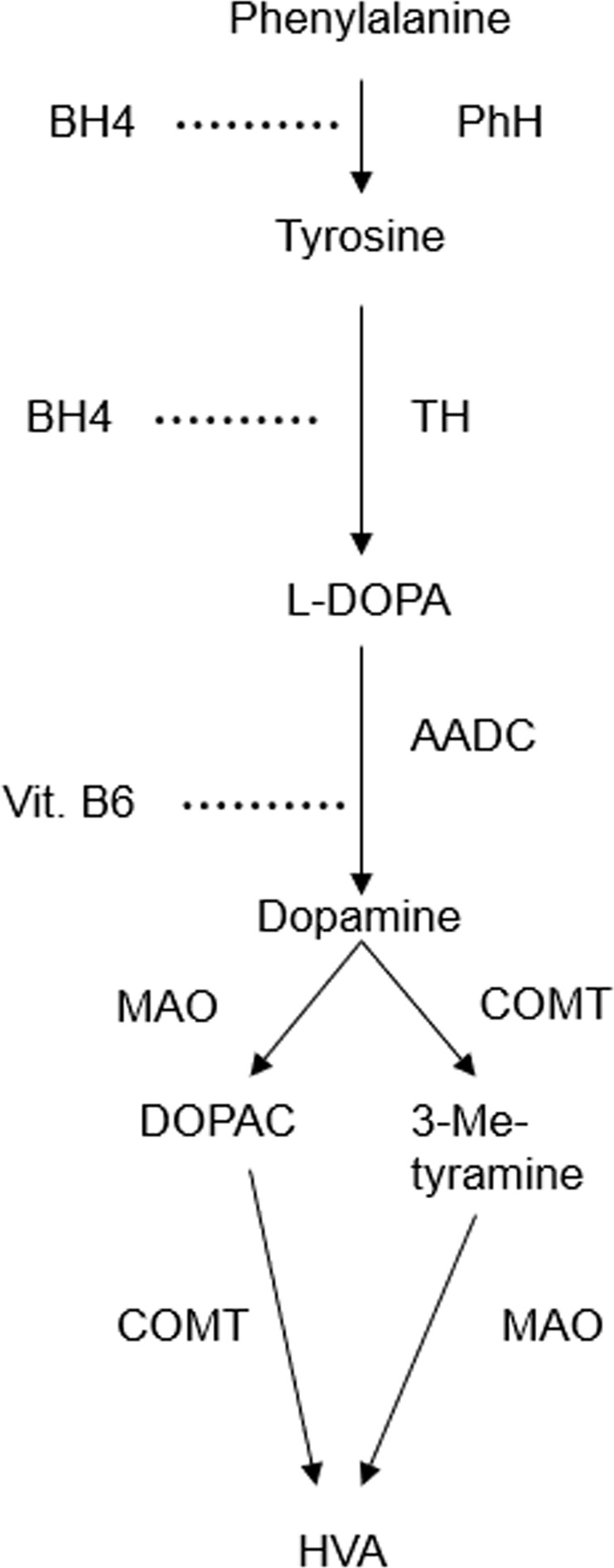
Production and metabolism of dopamine. Levodopa is produced in a two-step enzymatic reaction by conversion of the amino acid phenylalanine into l-tyrosine by the enzyme phenylalanine hydroxylase (PhH), and further into L-tyrosine by the enzyme tyrosine hydroxylase (TH). It is a direct precursor to the neurotransmitter dopamine (3,4-dihydroxyphenethylamine) by the action of the enzyme aromatic l-amino acid decarboxylase (AADC). Further breakdown of dopamine into 3-methoxy-tyramine, 3,4-dihydroxyphenylacetic acid (DOPAC) and homovanillic acid (HVA) is mediated by the enzymes catechol-O-methyltransferase (COMT) and monoamine oxidase (MAO)^5^. Vitamin B6 (vit. B6) is a co-factor for AADC; tetrahydrobiopterin (BH4) is a co-factor for both PhH and TH.

Since a dopamine deficit in the striatum is the neurochemical hallmark of PD, pharmacological substitution of dopamine is an important treatment modality. However, dopamine itself does not cross the blood-brain barrier (BBB) because of its high polarity and a lack of active transport proteins^11^. Its precursor levodopa, in contrast, is transported across the BBB by a selective transporter (L-type amino acid transporter 1, LAT1)^11^. Levodopa (l-3,4-dihydroxyphenylalanine, l-DOPA) is an amino acid which is naturally present in the human body. A diagram of its metabolism is provided in Fig. 1.

After oral ingestion and passage through the stomach, levodopa is absorbed by a saturable facilitated transport system for large neutral amino acids (LNAA)^12^, mostly in the duodenum and proximal jejunum^13^. In the intestinal mucosa, AADC converts levodopa into dopamine. Although active in the intestine, AADC is also found in the circulation and in other organs, primarily the kidneys, liver and brain^5^.

Due to premature metabolization and incomplete passage of the BBB, as little as 1–3% of levodopa reaches the brain if administered as monotherapy^14^. Combining levodopa with a peripheral decarboxylase inhibitor (PDI) such as carbidopa or benserazide, which inhibit the enzyme AADC, greatly increases the amount of levodopa available to the brain, and decreases the required oral dose of levodopa by 80%^15^.

## Primary and secondary non-responders

A gratifying and sustained levodopa response is a supportive criterion for the diagnosis of PD. According to the current clinical diagnostic criteria of the Movement Disorder Society (MDS), the “absence of observable response to high-dose levodopa despite at least moderate severity of disease” is regarded as an absolute exclusion criterion to the diagnosis^8^ (although there are caveats). While disease progression will necessitate progressively higher dosing (presumably due to increased nigrostriatal cell loss) and is accompanied by response fluctuations and levodopa-induced dyskinesias (due to altered pharmacodynamics), levodopa is expected to remain effective throughout the course of the disease^16^.

PD patients who manifest an absence or decrease of their clinical response to levodopa can be categorized into primary non-responders—those who never display a satisfactory response to levodopa despite adequate dosing – and secondary non-responders—those who, after an initially favorable response, gradually lose efficacy of levodopa over time and often experience diurnal fluctuations in spite of adequate dose escalation^15^. In addition to a lack of clinical benefit, another argument for non-response is the absence of response fluctuations (‘ON/OFF’ phenomena) and levodopa-induced dyskinesias after long-term treatment; both typically occur in 85% of patients after 10 years of levodopa treatment^9^.

For both forms of non-response, the first consideration should be *pseudoresistance*. As the name suggests, this pertains to the false impression that dopamine-sensitive symptoms or signs are (or become) resistant to levodopa^17^. Causes of pseudoresistance include insufficient dosing (because of dose-limiting side effects, or reluctance in either the patient or the physician to prescribe higher doses), and variations between individuals in pharmacokinetics and pharmacodynamics^17^. Also, not all PD features respond to the same extent to a given dosage of levodopa, and various combinations of levodopa responsiveness, levodopa resistance and levodopa-induced features can be seen for motor and non-motor symptoms^17^. For example, tremor typically responds less well to levodopa than bradykinesia, and in some individuals, tremor can be resistant to dopaminergic medication altogether, even in the presence of an otherwise gratifying levodopa response^17^.

A second option is that primary levodopa non-responders were in fact misdiagnosed, i.e., these individuals might not have PD to begin with, but could instead have a form of atypical parkinsonism or perhaps even have a completely different neurological condition that sometimes mimics parkinsonism, such as dystonia. However, autopsy-controlled studies in 1993 and 2020 demonstrated that 6.3–8.8% of autopsy-confirmed PD patients (which included individuals with “at least moderate severity of disease” as formulated in the MDS criteria) had nil to poor response to levodopa, and a further 16.8–22.5% had a moderate response (of which up to nearly half did not experience unequivocal wearing-off or dyskinesias)^9,10^. Another autopsy-controlled study in 2000 showed a poor *initial* levodopa response in 23% of PD patients^18^. In other words, true levodopa resistance does occur in people with autopsy-confirmed PD. An absent levodopa response, or a response perceived to be less than ‘marked’, may occur in up to a fifth of autopsy-confirmed PD patients and therefore does not exclude the diagnosis.

A further possible explanation for primary or secondary non-response includes delayed gastric emptying. This can result from PD itself, be caused by intake of meals^19^, be secondary to constipation^12,20^ (as a result of a mechanism that has been referred to as the ‘cologastric brake’)^21^ or result from levodopa treatment^22^. Delayed gastric emptying can significantly delay the passage of levodopa to the small intestine where it is absorbed, leading to a postponed effect (‘delayed-ON’) or even the absence of effect (‘no-ON’)^23–25^, as well as increasing the unpredictability of ‘ON-OFF’ fluctuations^19,22^. The proteinaceous contents of meals can alter levodopa pharmacokinetics as well. This so-called ‘protein competition’ occurs both in the gut and at the BBB. In the gut, dietary LNAAs compete with levodopa for the transport system, thereby reducing levodopa’s bioavailability^12^. At the level of the BBB, plasma long chain amino acids (which are elevated by dietary protein intake) compete with levodopa for the LAT1 transporter, delaying its entry into the brain and restricting the amount of levodopa available to the dopaminergic system^26^. Furthermore, infectious and altered metabolic states can decrease the permeability of the BBB to levodopa as well^27^.

**Fig. 2:**
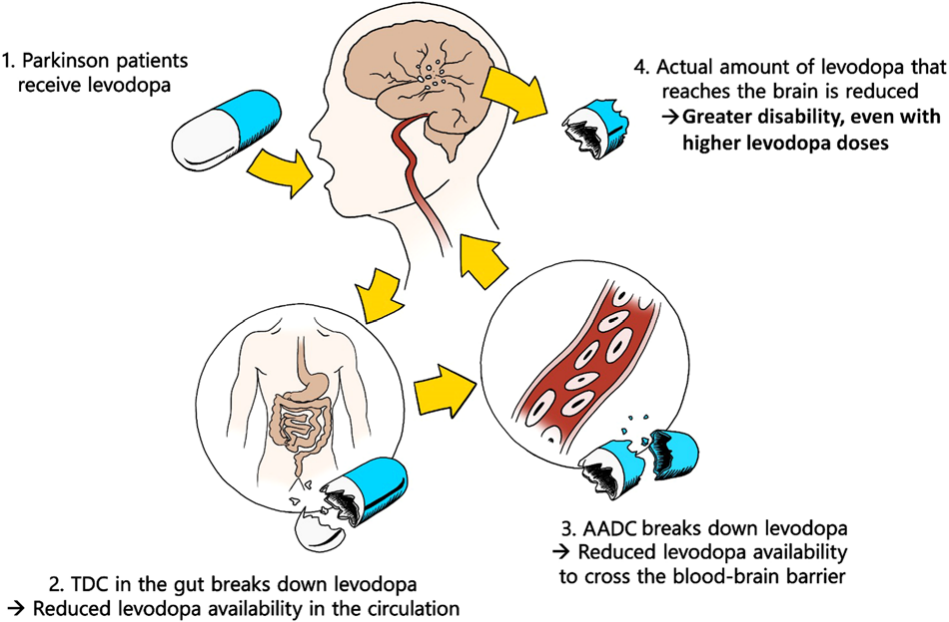
The two mechanisms of peripheral levodopa resistance. TDC tyrosine decarboxylase, AADC aromatic l-amino acid decarboxylase.

In addition to these factors, the differential diagnosis for primary non-response is also said to include a so-called idiopathic non-response in correctly diagnosed individuals. In many ways, however, this is an unsatisfactory explanation. We here offer two possible explanations for this idiopathic non-response, both of which can be seen as peripheral enzymatic blocks that are responsible for a peripheral conversion of levodopa into dopamine, thereby hampering the entry of levodopa into the brain. These mechanisms, which we collectively refer to as ‘peripheral levodopa resistance’, are summarized in Fig. 2 and further elaborated upon in the next sections.

## Mechanisms of peripheral levodopa resistance

### Small-intestinal bacterial overgrowth and TDC-producing bacteria as a cause of peripheral levodopa resistance

PD causes a range of gastrointestinal symptoms, some of which can predate the onset of motor symptoms by as many as 20 years^23^. Many of these symptoms result from delayed gastric emptying and decreased bowel motility, which in part result from α-synuclein-mediated degeneration of the myenteric and submucosal plexus^24^ and—in treated patients—from local effects of dopamine^25,28^. Delayed gastric emptying occurs in 70–100% of PD patients and results in symptoms such as nausea, early satiety and bloating^24,25^. Constipation is present in 50–90% of PD patients^23,25^ and is related to prolonged colonic transit time and pelvic floor dyssynergia, as well as medication-induced effects such as inhibition of colonic muscle contraction by anticholinergic and dopaminergic medication^23,28^.

Another important factor to consider is small-intestinal bacterial overgrowth (SIBO), which is defined as increased bacterial density in the small intestine caused by proximal migration of colonic bacteria^29^. Clinical correlates include bloating, constipation, excessive flatulence and diarrhea^30^. The prevalence of SIBO in PD has been estimated at 25–55%, with a higher prevalence in cohorts with more severe disease^30,31^. A recent meta-analysis found a pooled prevalence of SIBO in PD patients of 46%^32^. Risk factors include PD-related gastrointestinal dysmotility, longer disease duration and proton-pump inhibitor use^28^, as well as a concurrent diagnosis of diabetes mellitus or hypothyroidism^30^. In addition, there is a complex association between *Helicobacter pylori* (*H. pylori*) infection (occurring in a third of PD patients)^30^ and SIBO. Specifically, *H. pylori* infection itself causes slow gastrointestinal motility, and this is compounded by subsequent treatment with proton-pump inhibitors which are commonly used to treat the symptoms of *H. pylori* infection, but which are also a further risk factor for the development of SIBO^24^. As levodopa has pronounced regional differences in absorption (being mostly absorbed in the duodenum and proximal jejunum)^13,22^ it is highly dependent on small-intestinal absorption and the small-intestinal transit time^33^, both of which are adversely affected by SIBO. Through inflammation of the intestinal lining^30^, SIBO leads to malabsorption of levodopa. It also potentially leads to intestinal breakdown of levodopa. It is associated with worse motor function^31,34^ and with a high prevalence of unpredictable motor fluctuations (50–88%), with longer ‘OFF’ phases and more episodes of ‘delayed-ON’ and ‘no-ON’^30^. In addition, *H. pylori* can cause malabsorption and motor fluctuations independently of SIBO^24^. Recently, it was hypothesized that SIBO might even be implicated in the etiological cascade of PD and that its presence may contribute to the progression of PD through inflammation-induced synucleinopathy^35^. Therefore, the authors of that article consider that eradication of SIBO might be warranted in PD patients even in the absence of GI symptoms.

A variety of gut microbial changes have been reported in several studies in PD patients. The most consistently reported increased abundance is of the genera *Akkermansia* (12 studies), *Lactobacillus* (10 studies), and *Bifidobacterium* (4 studies); in contrast, the abundance of *Prevotella* is reported as *decreased* in 12 studies^36–39^. This change in gut microbiome composition may play a seminal role in a microbial pathway of levodopa resistance. In addition, it might be a prerequisite for the development of the so-called *body-first* phenotype of PD^40^ (consistent with the bottom*-*up hypothesis, where the pathophysiological process may start in the gut, and subsequently spread via the vagal nerve to ultimately reach the brain)^4^.

Tyrosine decarboxylase (TDC) is an enzyme which normally digests dietary tyrosine. However, TDC, produced by certain gut bacteria, can also decarboxylate levodopa, reducing its bioavailability. This conversion is not inhibited by AADC inhibitors such as carbidopa and benserazide^41^. A positive association has been found between bacterial TDC gene expression in stool samples and daily levodopa dose requirement, and levodopa levels in plasma correlate negatively with bacterial TDC gene expression in the jejunum^41^. In addition, in a 2021 study, dosage increases of catechol-O-methyltransferase (COMT) inhibitors and dopamine agonists were associated with increased TDC gene abundance, whereas the reverse was true for monoamine oxidase inhibitor dose^42^. The direction of causality—if any—is unclear, although disease duration was corrected for. The most important TDC-producing bacteria in the human gut are *Enterococcus faecalis*, *Enterococcus faecium*, *Lactobacillus brevis* and, to a lesser extent, *Providencia rettgeri*^43^. Indeed, *Lactobacilllus* is one of the bacterial genera of which an increased abundance has been reported in PD patients. As the micro-organisms involved in SIBO originate from the colonic flora, it would be reasonable to hypothesize that *Lactobacillus* is present in the small-intestinal flora of PD patients with SIBO. Moreover, a PD-associated altered microbiome might not be a prerequisite for overgrowth of TDC-producing bacteria, as was recently demonstrated in a significant proportion of healthy controls in whom TDC gene was detectable in feces as well^42^. The increase in TDC gene abundance over time was significantly higher in PD patients, however^42^. Remarkably, that study also suggested that *less* constipation was associated with *increased* TDC gene abundance^42^, which is in contradiction with the theoretical model in which constipation facilitates bacterial overgrowth. Luminal dopamine produced by bacterial species, such as enterococci, can further affect gut motility and form a perpetuating factor in SIBO^28,41^. Tyramine, a product of bacterial TDC, can also impair gut motility^28^.

**Fig. 3:**
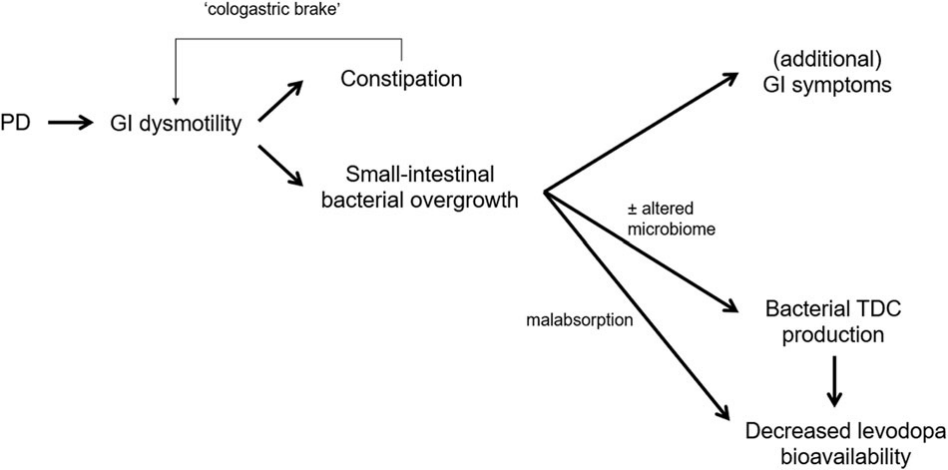
Relationship between Parkinson’s disease, intestinal factors and decreased levodopa bioavailability. Relationship between Parkinson’s disease (PD), gastrointestinal (GI) function, constipation, small-intestinal bacterial overgrowth, tyrosine decarboxylase (TDC) production, and reduced levodopa efficacy.

The relationship between the above-mentioned factors is visualized in Fig. 3.

### Systemic induction of AADC enzyme activity as a cause of peripheral levodopa resistance

We recently demonstrated that administration of levodopa concurrently with a PDI can paradoxically induce blood AADC enzyme activity^44^, a finding described only once previously in the 1980s^45^. In this study by our group, AADC enzyme activity was higher (by ~80%) in patients using levodopa/PDI, regardless of the diagnosis and after adjustment for disease duration. The likelihood of elevated AADC activity was also higher in patients using higher daily levodopa doses. Increased AADC activity implies a shorter peripheral half-life of levodopa (increasing the likelihood of response fluctuations) and a reduction of the amount of levodopa available to the brain. Patients with higher AADC activity were also more likely to use additional medication, such as COMT inhibitors and dopamine agonists. This suggests that not only did they have altered levodopa pharmacokinetics, but levodopa monotherapy also failed to result in adequate symptom control.

The induction of AADC is thought to be caused by a feedback mechanism rather than general liver enzyme induction, as increased AADC activity was not found in patients using known liver enzyme inducing drugs such as phenytoin^45^. The effect was previously not observed in patients using levodopa without PDI^45^. The underlying physiology of this feedback mechanism has yet to be explored. AADC expression may be regulated both at the transcriptional and translational level^46^. Alternative splicing and promotor usage have both been proposed as mechanisms of, potentially tissue-specific, AADC transcriptional regulation machineries^47,48^. Dopamine antagonists are among many compounds that may regulate AADC mRNA expression^46^. AADC phosphorylation, which may increase its activity^46^, can be induced by activators of cAMP-dependent protein kinase A (PKA), by activators of protein kinase C or by activators of protein phosphatase inhibitors (see ref. ^46^ for an overview). A number of hypotheses could be formulated on how levodopa/PDI use may lead to increased AADC activity. Stimulation of dopamine receptors in the brain (such as induced by levodopa therapy) may downregulate cerebral AADC synthesis^46^. As co-administration of a PDI results in relatively low serum levels of dopamine, peripherally the lack of dopamine receptor stimulation might lead to upregulation of AADC synthesis. In this scenario, the fact that AADC induction was previously not observed in patients using levodopa without a PDI^45^ may not indicate that the induction is caused by the PDI itself. Rather, the high rate of peripheral conversion to dopamine resulting from levodopa monotherapy would mean that the aforementioned understimulation of peripheral dopamine receptors does not occur, and thus AADC is not induced. An ostensible discrepancy between central downregulation and peripheral upregulation may also reflect selective, tissue-specific, regulation^46^. Alternatively, PDIs might themselves increase AADC expression and/or activity. Chronic use of levodopa/PDI can cause vitamin B6 depletion^49^. One cause of this may be the irreversible binding of PDIs to the active form of vitamin B6^49^, another might be saturation of the methionine cycle that facilitates levodopa metabolization by COMT^50^. This vitamin B6 depletion may (possibly through resultant dysfunctional methionine metabolism) increase AADC expression or promote its phosphorylation. Further research will be necessary to elucidate the mechanism(s) involved.

## Further research and possible therapeutic interventions

Recognition of these two peripheral blockades that may hamper levodopa’s efficacy could have relevant consequences for the management of individuals with PD who experience a less-than-expected clinical response to levodopa. After ruling out pseudoresistance and aggressively treating constipation as a possible cause of levodopa malabsorption (through constipation-induced delayed gastric emptying), multiple strategies can be employed to combat peripheral levodopa resistance. The first strategy entails approaches aimed at correcting SIBO and the reduction of bacterial TDC expression levels, thereby reducing the enzymatic conversion of levodopa into dopamine within the gut. The second strategy is to employ oral dopamine agonists, which are not dependent on either TDC or AADC. The third strategy aims to bypass the unreliable gastrointestinal system, using parenteral pharmacotherapeutic strategies. This bypass approach also offers a solution for individuals whose levodopa resistance results at least in part from AADC induction. We also offer some directions for future research, aiming to provide a further evidence base for both the presumed mechanisms of peripheral resistance and the recommended therapeutic solutions.

### Treatment of SIBO

The most obvious first-line treatment for SIBO would be antibiotic eradication. Small trials that examined SIBO eradication^29,30^ tested rifaximin, a non-absorbable oral antibiotic covering a wide range of Gram-positive and Gram-negative bacteria (including *Akkermansia* spp, *Enterococcus* spp, *Lactobacillus* spp and *Bifidobacterium* spp, although *P. rettgeri* is largely resistant)^51–53^. The results showed a reduced ‘OFF’-time, reduced ‘delayed-ON’, reduced wearing-off and reduced ‘no-ON’, while the eradication therapy was well-tolerated^30^. A drawback of rifaximin relates to its high costs in some markets, including the United States (over $850 for a 7-day course of 550 mg t.i.d.)^54^. Given the reported prevalence of SIBO in PD, and the rapidly growing prevalence of PD itself, the financial burden to national health care would be substantial, although some and perhaps even most of the treatment costs might be offset by the resulting more gratifying response to levodopa, leading to less disability, fewer disease complications and presumably fewer hospital visits.

Another non-absorbable antibiotic that could theoretically be employed is vancomycin, a glycopeptide antibiotic that is effective against Gram-positive bacteria. Although there are no published articles about the use of this agent for the treatment of SIBO, the drug is used extensively for the treatment of *Clostridioides difficile* infection. Limited experience exists for the use of oral vancomycin for the treatment of chronic idiopathic constipation^55–57^ and for the alteration of human intestinal microbiota^58^. An obvious advantage of this antibiotic would be the greatly-reduced cost as compared to rifaximin in some markets, including the United States, totaling just under $150 for a 14-day course (250 mg t.i.d.)^54^. (It should be noted that the reverse is true in some other jurisdictions, such as the Netherlands, where a course of rifaximin is 25% of the cost of a course of oral vancomycin.)^59^ Also, the fact that oral vancomycin is not absorbed into blood has clear advantages with regard to e.g., side effects, allergies and the development of antibiotic resistance. In addition, in contrast to oral vancomycin, broad-spectrum absorbable antibiotic agents have a propensity to cause diarrhea or loose stools^55^.

There are, however, a number of drawbacks to the use of vancomycin that warrant consideration:

- Antibiotic sensitivity. *Enterococcus* spp, being Gram-positive, are generally sensitive to vancomycin (with the exception of vancomycin-resistant enterococci). However, *Lactobacillus* spp, of which *L. brevis* is TDC-producing, are intrinsically resistant to vancomycin^60^, and two studies in fact demonstrated an *increase* in the abundance of *Lactobacillus* spp after vancomycin treatment^58,61^. *Bifidobacterium* spp are generally vancomycin-sensitive^62^, but *Akkermansia*, being a Gram-negative genus, is by definition resistant to vancomycin;
- Further disturbance of the gut microbiome, potentially giving free rein to bacterial species associated with infections. Even a single, 7–14 day course of oral vancomycin has been demonstrated to induce both short- and long-term adverse changes in the richness of the gut microbiome^58,61,63–65^, resulting in a relative abundance of pathogenic *Proteobacteria* species including *Klebsiella, Escherichia* and *Shigella*^58,61,64,65^;
- Promotion of antibiotic resistance and intestinal colonization by vancomycin-resistant enterococci^58^. In a subset of patients receiving vancomycin for *Clostridioides difficile* eradication, secondary infection with vancomycin-resistant enterococci has been described^66–68^.

Given the complex interplay between *H. pylori* infection, SIBO and PD, ideally the employed antibiotic agent would eradicate *H. pylori* in addition to TDC-producing *Enterococcus* and *Lactobacillus spp*. The efficacy of rifaximin monotherapy for *H. pylori* eradication, however, is disappointing^69^, whereas the effect of vancomycin is non-existent (*H. pylori* being Gram-negative). The standard regimen for *H. pylori* eradication is a combination therapy which, depending on regional antibiotic resistance, entails either a triple therapy, consisting of a proton-pump inhibitor, clarithromycin and amoxicillin, or a quadruple therapy, comprised of a proton-pump inhibitor, bismuth, metronidazole and tetracycline^70^. A recent small (*n* = 67) randomized controlled trial failed to show benefit of *H. pylori* eradication in PD patients^71^. However, a 2018 meta-analysis including case-control studies and cohort studies as well as randomized controlled trials (*n* = 90), demonstrated that *H. pylori* eradication in PD patients significantly reduced motor symptoms^72^. Whether additional *H. pylori* eradication in antibiotically treated SIBO patients would have added benefit on gastrointestinal and PD symptoms could be a subject of future research.

Advantages and disadvantages of the various antibiotic choices will have to be carefully weighed when designing future studies to further test their efficacy in reversing SIBO and, importantly, restoring levodopa efficacy. Trials of antibiotic treatment in a selected sample of PD patients who manifest both levodopa non-response and symptoms of SIBO will be necessary.

Besides antibiotics, another treatment that has been suggested for bacteria-related reduction of levodopa bioavailability is (S)-α-fluoromethyltyrosine (AFMT), which prevents L-dopa decarboxylation by TDC through the disabling of pyridoxal-5′-phosphate^43^. As far as we know, there is no documented real-life experience with this intervention as of yet. A further treatment modality that might warrant consideration, given the altered gut microbiome in PD patients which ultimately might contribute to the development of SIBO, is the administration of probiotics. In several studies of oral probiotics in constipated PD patients, a significant improvement was seen in the number of bowel movements, stool consistency and quality of life^73,74^. Whether this might also be effective for the treatment or prevention of SIBO in PD patients could be a subject of further research. Given the paucity of trials thus far, it is not yet clear which probiotic strains would be likely to be effective. The available studies used species belonging to the genera *Bifidobacterium*, *Enterococcus*, *Lactobacillus* and *Streptococcus*^73–75^. It stands to reason that any probiotic preparation used in an attempt to treat or prevent SIBO in PD should exclude strains known to be TDC-producing.

### ‘Bypassing the enzymes’

TDC and AADC are involved in the metabolization of levodopa, but not that of dopamine agonists. In persons in whom increased enzyme activity is the cause of levodopa resistance, dopamine agonists could be employed. Oral treatment with a dopamine receptor agonist might be the most straightforward solution. It may, however, fall short of expectations in patients who also have intestinal malabsorption, such as in SIBO or in delayed gastric emptying. Indeed, it has been suggested that delayed gastric emptying negatively influences absorption of the dopamine agonist ropinirole^76^. Pramipexole is absorbed into the blood by organic cation transporters in the brush border membrane of the jejunum^77^. It would be reasonable to presume that SIBO-related inflammation of the jejunal lining negatively affects pramipexole absorption. In a case report, an ileostomy patient with idiopathic parkinsonism failed to respond not only to oral levodopa but also to oral pramipexole, whilst showing marked improvement in response to transdermal rotigotine^78^.

### ‘Bypassing the gut’

In persons in whom the above-mentioned options fail to improve PD symptoms (or only for a short while), a potentially viable option could be *bypassing the gut* using non-enteral dopaminergic medication. Some of these approaches still include levodopa, such as an inhalable powder-form levodopa/carbidopa preparation^79^ which recently gained marketing approval by US regulators. This approach would be a possible solution for patients experiencing levodopa resistance due to excessive TDC production in the gut and/or SIBO-related malabsorption, but would not be helpful for patients whose resistance is largely explained by AADC induction. For the latter group, a viable option would be treatment with a parenteral dopamine receptor agonist. Currently available options include subcutaneous apomorphine and transdermal rotigotine^80^. A newly-developed sublingual apomorphine film^81^ recently gained marketing approval by US regulators.

**Fig. 4:**
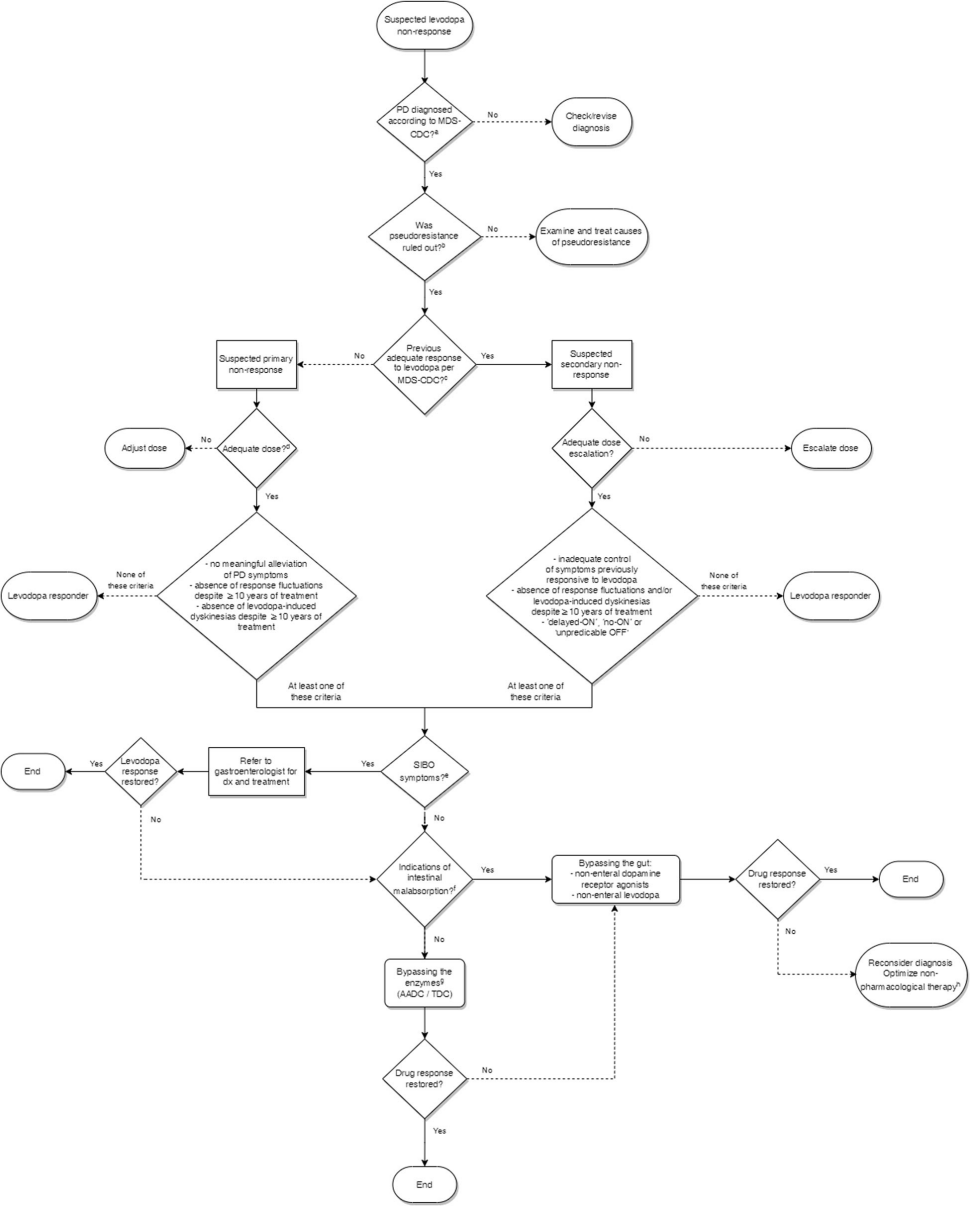
Flowchart detailing a possible diagnostic and treatment approach to suspected levodopa non-response. a/c See Postuma et al^8^. b Rule out insufficient dosing, trial a dosage increase, consider add-on therapy for symptomatic relief of dose-limiting side effects (e.g., domperidone for peripheral dopaminergic effects such as nausea, amantadine for dyskinesias) - Is the resistant symptom known to be less-responsive to dopaminergic medication (axial symptoms, tremor)? - Rule out & treat underlying gastroparesis and constipation - Check for, and phase out, dopamine receptor blocking or dopamine depleting co-medication. d At least 600 mg levodopa/day during at least 1 month^8^. e Bloating, constipation, excessive flatulence and/or diarrhea. f Such as residual inflammation of intestinal lining despite SIBO treatment, delayed gastric emptying. g Using oral dopamine receptor agonists. h If no effect, consider wrong diagnosis (primary non-response) or severe nigrostriatal degeneration in the context of disease progression (secondary non-response). Non-pharmacological therapy includes paramedic care and deep brain stimulation in case of symptoms that may respond better to DBS than to dopaminergic medication (tremor, dystonia, dyskinesia). PD Parkinson’s disease, MDS-CDC Movement Disorder Society Clinical Diagnostic Criteria for Parkinson’s Disease, SIBO small-intestinal bacterial overgrowth.

An overview of these approaches and their possible indications is provided in Table 1. A flowchart detailing a possible diagnostic and treatment approach is provided in Fig. 4. Fecal TDC gene measurement and serum AADC activity assay were not yet included in the algorithm. As of yet, they are not widely available for clinical use and indeed, it has yet to be determined whether these biomarkers are useful to guide clinical decision-making. All these approaches should be the subject of future trials.

**Table 1:** Overview of possible therapeutic approaches to combat various causes of peripheral levodopa resistance.

	Causes of peripheral levodopa resistance		
	Bacterial TDC production	Peripheral AADC induction	SIBO-related malabsorption of drugs
Oral dopamine agonist	+	+	
Parenteral dopaminergic therapy	+	+ (except levodopa-containing preparations)	+
Antibiotic therapy	+		+

*TDC* tyrosine decarboxylase, *AADC* aromatic L-amino acid decarboxylase, *SIBO* small-intestinal bacterial overgrowth.

Obviously, correct patient selection will be key to the success of any treatment. Patients eligible for SIBO eradication, enzyme-bypassing and/or gut-bypassing medication should be selected by a combination of (primary or secondary) levodopa non-response, gastrointestinal symptoms, and perhaps biomarkers such as bacterial TDC gene in feces and AADC activity in serum. To screen for the presence and severity of gastrointestinal symptoms, the newly-devised Gastrointestinal Dysfunction Scale for Parkinson’s Disease (GIDS-PD)^82^ might provide the necessary guidance.

### Final remarks

The efficacy of levodopa, the mainstay of pharmacological treatment for the majority of PD patients, depends on an adequate and predictable quantity reaching the brain. Malabsorption and peripheral breakdown of levodopa can restrict its cerebral availability in a subset of patients. This may lead to unexpected therapy failure in patients who should normally respond to levodopa. Bacterial TDC production in the small intestine and paradoxical AADC induction are two mechanisms underlying peripheral levodopa resistance which have received limited attention thus far, but which might be relevant to a sizeable subgroup of PD patients. An important focus of future research should be the identification and tailored treatment of these patients.

### Reporting summary

Further information on research design is available in the Nature Research Reporting Summary linked to this article.

## Supplementary information

Reporting Summary
